# NFAT5 Is Activated by Hypoxia: Role in Ischemia and Reperfusion in the Rat Kidney

**DOI:** 10.1371/journal.pone.0039665

**Published:** 2012-07-02

**Authors:** Sandra Villanueva, Cristian Suazo, Daniela Santapau, Francisco Pérez, Mariana Quiroz, Juan E. Carreño, Sebastián Illanes, Sergio Lavandero, Luis Michea, Carlos E. Irarrazabal

**Affiliations:** 1 Laboratory of Molecular Physiology, Faculty of Medicine, Universidad de Los Andes, Santiago-Chile; 2 Laboratory of Integrative Physiology, ICBM, Universidad de Chile, Santiago-Chile; 3 Centro de Estudios Moleculares de la Célula, Facultad de Medicina, Universidad de Chile, Santiago-Chile; 4 Facultad de Ciencias Químicas y Farmacéuticas, Universidad de Chile, Santiago-Chile; 5 Cardiology Division, Department of Internal Medicine, University of Texas Southwestern Medical Center, Dallas, Texas, United States of America; Universidade de Sao Paulo, Brazil

## Abstract

The current hypothesis postulates that NFAT5 activation in the kidney's inner medulla is due to hypertonicity, resulting in cell protection. Additionally, the renal medulla is hypoxic (10–18 mmHg); however there is no information about the effect of hypoxia on NFAT5. Using *in vivo* and *in vitro* models, we evaluated the effect of reducing the partial pressure of oxygen (PO_2_) on NFAT5 activity. We found that 1) Anoxia increased NFAT5 expression and nuclear translocation in primary cultures of IMCD cells from rat kidney. 2) Anoxia increased transcriptional activity and nuclear translocation of NFAT5 in HEK293 cells. 3) The dose-response curve demonstrated that HIF-1α peaked at 2.5% and NFAT5 at 1% of O_2_. 4) At 2.5% of O_2_, the time-course curve of hypoxia demonstrated earlier induction of HIF-1α gene expression than NFAT5. 5) siRNA knockdown of NFAT5 increased the hypoxia-induced cell death. 6) siRNA knockdown of HIF-1α did not affect the NFAT5 induction by hypoxia. Additionally, HIF-1α was still induced by hypoxia even when NFAT5 was knocked down. 7) NFAT5 and HIF-1α expression were increased in kidney (cortex and medulla) from rats subjected to an experimental model of ischemia and reperfusion (I/R). 7) Experimental I/R increased the NFAT5-target gene aldose reductase (AR). 8) NFAT5 activators (ATM and PI3K) were induced *in vitro* (HEK293 cells) and *in vivo* (I/R kidneys) with the same timing of NFAT5. 8) Wortmannin, which inhibits ATM and PI3K, reduces hypoxia-induced NFAT5 transcriptional activation in HEK293 cells. These results demonstrate for the first time that NFAT5 is induced by hypoxia and could be a protective factor against ischemic damage.

## Introduction

Concentration of urine by the mammal kidney requires an osmolality gradient from cortex to medulla. In conditions of free access to water, the renal cortex has an osmolality of about 300 mOsm, while osmolality in the renal papilla osmolality is approximately 800 mOsm. During maximal antidiuresis, medullary osmolality can reach 1200–1800 mOsm. Such high osmolality threatens renal papillary cells, and protective mechanisms are required for their survival [1]–[3].

NFAT5 (Nuclear factor of activated T-cells) is a member of the Rel family of transcriptional activators, which includes nuclear factor κB (NFκB). This factor has been identified as the transcription factor necessary for survival of renal cells in the challenging conditions of renal medulla [4]–[6]. NFAT5-knockout mice are embryonically lethal, and the surviving NFAT5-null mice have profound and progressive atrophy of the renal medulla [7]. Transgenic (Tg) mice overexpressing NFAT5dn (dominant negative form of NFAT5) show impaired urine concentration, progressive atrophy of the renal medulla, cortical thinning, and severe hydronephrosis [8].

NFAT5 is rapidly activated by hypertonicity. In cells cultured at 300 mOsm, NFAT5 is present in the nucleus and cytoplasm, but a change to hypertonic medium triggers NFAT5 translocation to the nucleus and upregulation of NFAT5 gene expression [9]–[13]. In the rat kidney, nuclear localization of NFAT5 decreases after water loading and increases after dehydration [14]. The activation of NFAT5 by hypertonic stress results in the induction of several genes implicated in osmotic tolerance, such as aldose reductase (AR) [15], [16]. The NFAT5 target genes contain at least one osmotic response element (ORE) consensus [17], [18] and AP-1 site [19].

There are positive and negative upstream molecular regulators of the tonicity-dependent activation of NFAT5 transactivating activity: RNA helicase A [20]; epidermal growth factor receptor (EGFR) [21]; cAMP-dependent kinase (PKA) [22]; p38 mitogen-activated protein kinase (MAPK) [23]; Fyn, a member of the SRC family of non-receptor, cytoplasmic protein tyrosine kinases [23]; Ataxia Telangiectasia Mutated (ATM) [24]; phosphatidyl 3-kinase Class IA (PI3K-IA) [25]; eNOS-NO system [26]; and PLCγ1 [27]. Experiments using HEK293 and Jurkat cells suggest that PI3K class IA is upstream of ATM in high NaCl-induced activation of NFAT5 [25].

In addition to the corticomedullary osmolality gradient, the kidney has a corticomedullary oxygen gradient: the renal cortex has an arterial partial pressure of oxygen (PaO_2_) of 45–50 mmHg, while the deepest zones of the renal medulla have a pressure of only 10–18 mmHg [3]. In fact, the renal papilla receives less than 1% of total renal blood flow [2]. Therefore, medullary cells must also have a protective response to hypoxia.

The current hypothesis postulates that NFAT5 presents tonicity-dependent activation stimulated by oxidative stress [28], [29]. The urine concentrating mechanism in the kidney implies an increased osmolality, associated with low PO_2_, allowing reactive oxygen species (ROS) to increase [30]. Moreover, there is evidence suggesting that ATM and PI3K (activators of NFAT5) are activated during hypoxia in cancer cells [31], [32]. We hypothesize that low oxygen concentration induces NFAT5 activation. To study the effect of anoxia/hypoxia (0–5% O_2_) on NFAT5 expression and activity, we used primary cultures of inner medullary collecting duct (IMCD) and HEK293 cells, growing in isotonic and hypertonic media. We also analyzed the effect of hypoxia on cell death in NFAT5-knockdown cells. Pharmacological inhibition of NFAT5-activators (ATM and PI3K) was used to study the role of these kinases on NFAT5 induction by hypoxia. Additionally, we tested the *in vivo* effect of ischemia and reperfusion (I/R) on NFAT5 activity in an experimental model of renal I/R in the rat. In I/R kidneys, we measured mRNA and protein abundance of NFAT5, HIF-1α, one of its downstream genes (aldose reductase, AR), and two of its upstream activators (ATM and PI3K). Our results showed that NFAT5 is activated *in vitro* and *in vivo* by hypoxia and ischemia/reperfusion.

## Results

### Effect of hypoxia on NFAT5

**Figure 1:**
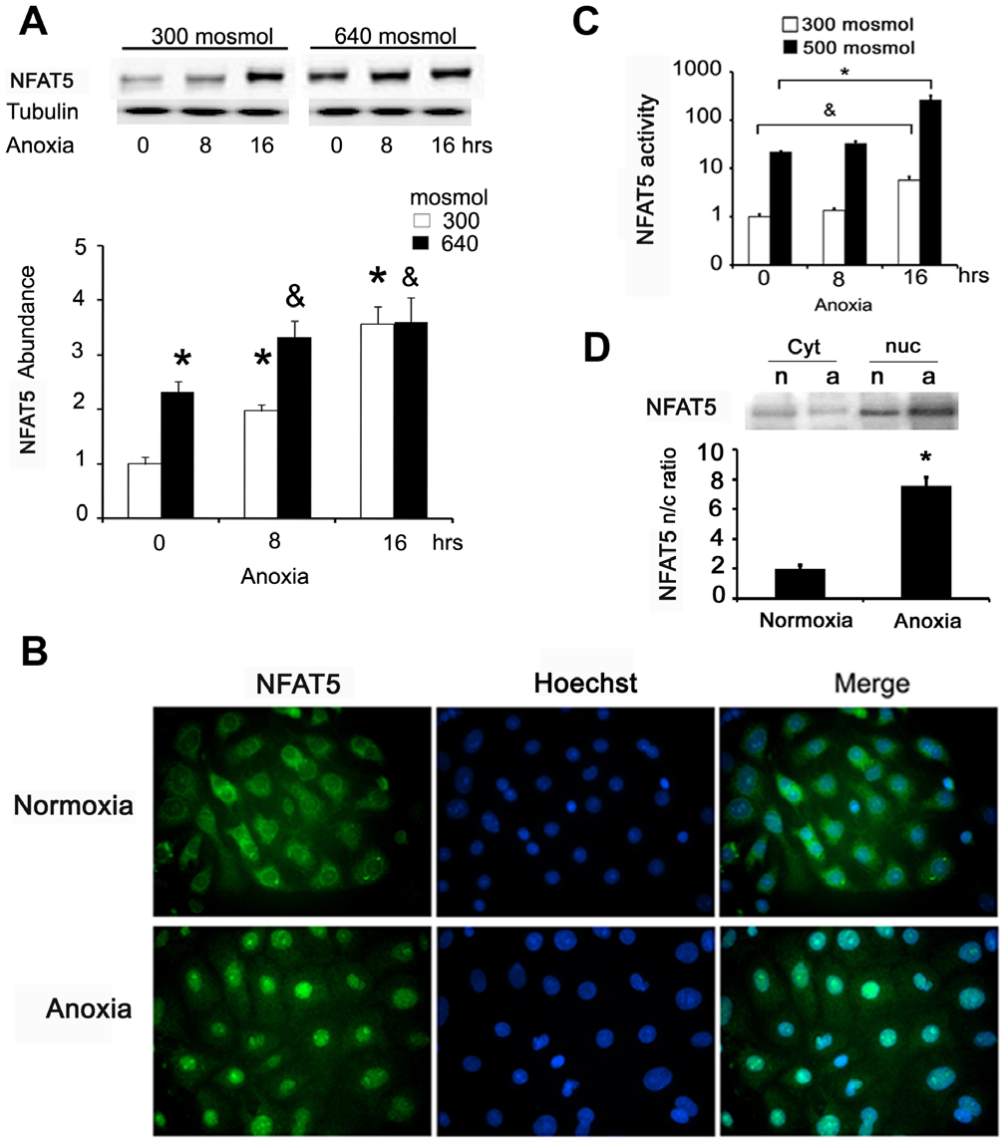
Anoxia increases NFAT5 protein abundance and promotes nuclear translocation. **A.** Rat primary IMCD cells in isotonic (300 mOsM) or hypertonic (640 mOsM) medium were exposed to anoxia (replacement of O_2_ by N_2_) for 0, 8, or 16 hrs. We prepared total protein homogenates and determined NFAT5 protein abundance by Western blot. A representative picture is shown in the upper section and the graph shows mean ± SEM. * or & P≤0.05; n = 5. (*vs. 300 mosmol/normoxia and & *vs.* 640 mosmol/normoxia). **B.** NFAT5 cellular distribution after 2 hrs of anoxia evaluated in primary IMCD cells by immunofluorescence. Green = NFAT5 labelling (Alexa488); blue = nuclei (Hoechst 33258). **C.** HEK293 cells stably expressing ORE-X cultured at 300 mosmol or 500 mosmol by 16 hrs; during this time the cells were exposed for 0, 8 or 16 hrs to anoxia, and luciferase reporter assay was used to evaluate transcription activity; Bar graph represents Mean ± SEM. (* or &, P<0.05; n = 5). **D.** HEK293 cells cultured at 300 mosmol were exposed by 2 hrs to anoxia (a) or normoxia (n). Nuclear and cytoplasmatic fractions were separated by NE-PER and NFAT5 abundance was determined by Western blot. Bar graph represents Mean ± SEM. (* P<0.05; n = 5).

We analyzed the effect of anoxia (0% of O_2_) on the NFAT5 protein abundance in primary cultures of inner medullary collecting duct (IMCD) cells from rat kidney and HEK293 cells. We used the hypertonic condition as a positive control for NFAT5 activation [19]: 640 mosmol for primary IMDC cells (similar to the condition in kidney medulla of rats with free access to water) [1]. IMCD cells were cultured in isotonic (300 mOsM) or hypertonic (640 mOsM) media for 24 hrs. As expected, hypertonicity increased NFAT5 abundance in primary IMCD cells (Figure 1A, 0 hrs anoxia). In isotonic media, anoxia induced NFAT5 protein abundance to 120–250% over the control, after 8 and 16 hrs of anoxia, respectively (Figure 1A). In the hypertonic condition (24 hrs), 8 hrs of anoxia caused an additional induction of NFAT5 protein abundance (Figure 1A, 8 hrs anoxia).

We tested the effect of anoxia on NFAT5 activation. First, we evaluated its nuclear translocation in primary IMCD cells under anoxia. Using fluorescence microscopy, we observed that NFAT5 nuclear localization was induced after 2 hrs of anoxia (Figure 1B). Next, we evaluated the transcriptional activity of NFAT5 using HEK293-OREX cells (stably expressing the reporter gene OREX) [19]. As in our previous studies with HEK293 cells [19], we used 500-mosmol hypertonic medium as a positive control for NFAT5 activation. In an isotonic medium, 16 hrs of anoxia increased the transcriptional activity of NFAT5 4.6-fold (Figure 1C). In hypertonic medium, anoxia (16 hrs) increased the transcriptional activity of NFAT5 9.6-fold as compared to the normoxia condition (21% oxygen and 500 mosmol; Figure 1C). In anoxia, we observed 25% of LDH release during 16 hrs of anoxia, suggesting cell death by necrosis (data not shown). These results suggest that anoxia induced the NFAT5 transcriptional activity independently of tonicity. Using Western blot analysis of nuclear and cytosolic proteins fractions obtained from HEK293 cells, we corroborated nuclear translocation of NFAT5 induced by anoxia (Figure 1D), similar to what we observed in IMCD cells (Figure 1B).

**Figure 2:**
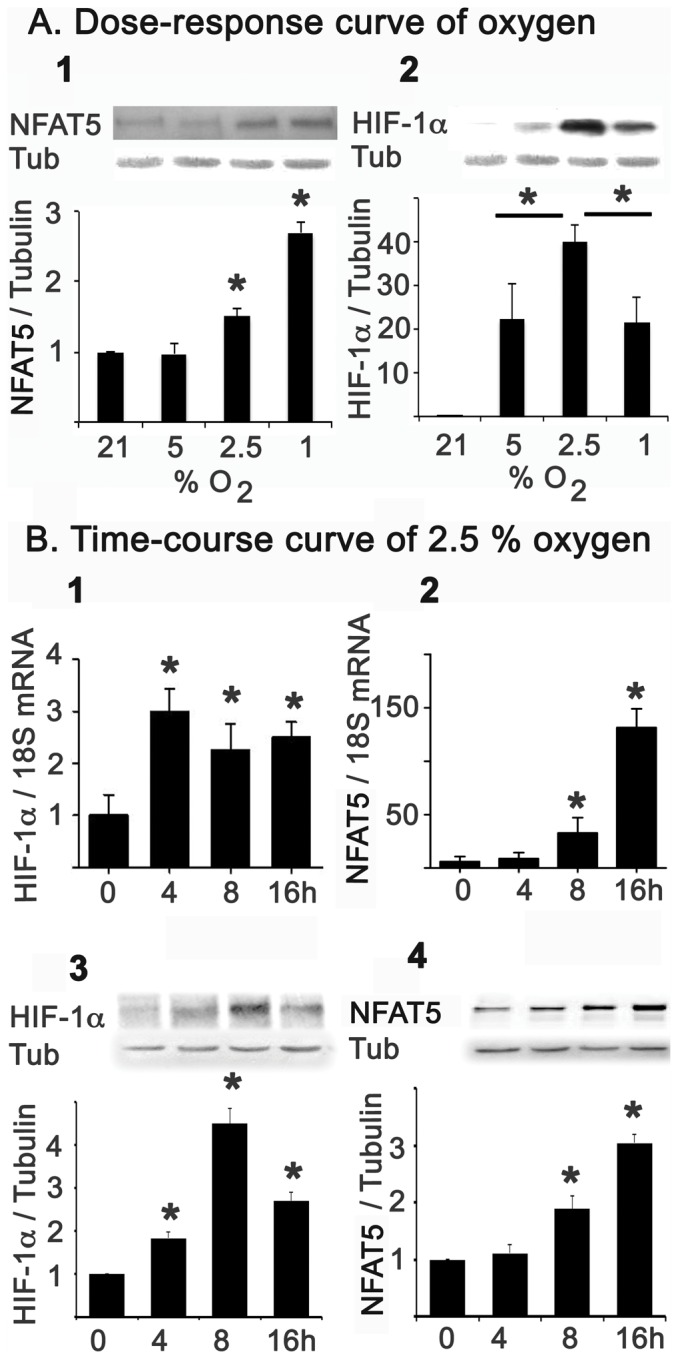
Hypoxia induces NFAT5 in cell culture. A. HEK293 cells cultured at 300 mosmol were subjected to dose-response curve of hypoxia (21, 5, 2.5 and 1% O_2_). NFAT5 (A1) and HIF-1α (A2) protein abundance was determinate by Western blot. B. Using 2.5% of PO_2_, cells were exposed for 0, 4, 8, and 16 hrs to analyse the HIF-1α gene expression by qRT-PCR (B1) and Western blot (B3). NFAT5 gene expression was also determined by qRT-PCR (B2) and Western blot (B4). Protein abundance and mRNA were normalized by tubulin (Tub) and 18S, respectively. Bar graph represents Mean ± SEM. *, P<0.05; n = 5.

To gain some insight into the potential mechanisms leading to NFAT5 activation by low oxygen, we exposed HEK293 cells to several hypoxia conditions (1.0, 2.5, 5.0 or 21% O_2_). In these experiments, we evaluated the gene expression of NFAT5 and Hypoxia-inducible factor-1 alpha (HIF-1α; Figure 2A). HIF-1α induction was observed starting from 5% O_2_, peaking at 2.5% O_2_. However, NFAT5 protein abundance induction was observed only when O_2_ concentration was decreased to 2.5% and was maximal when cells were at 1% O_2_ (Figure 2A).

**Figure 3:**
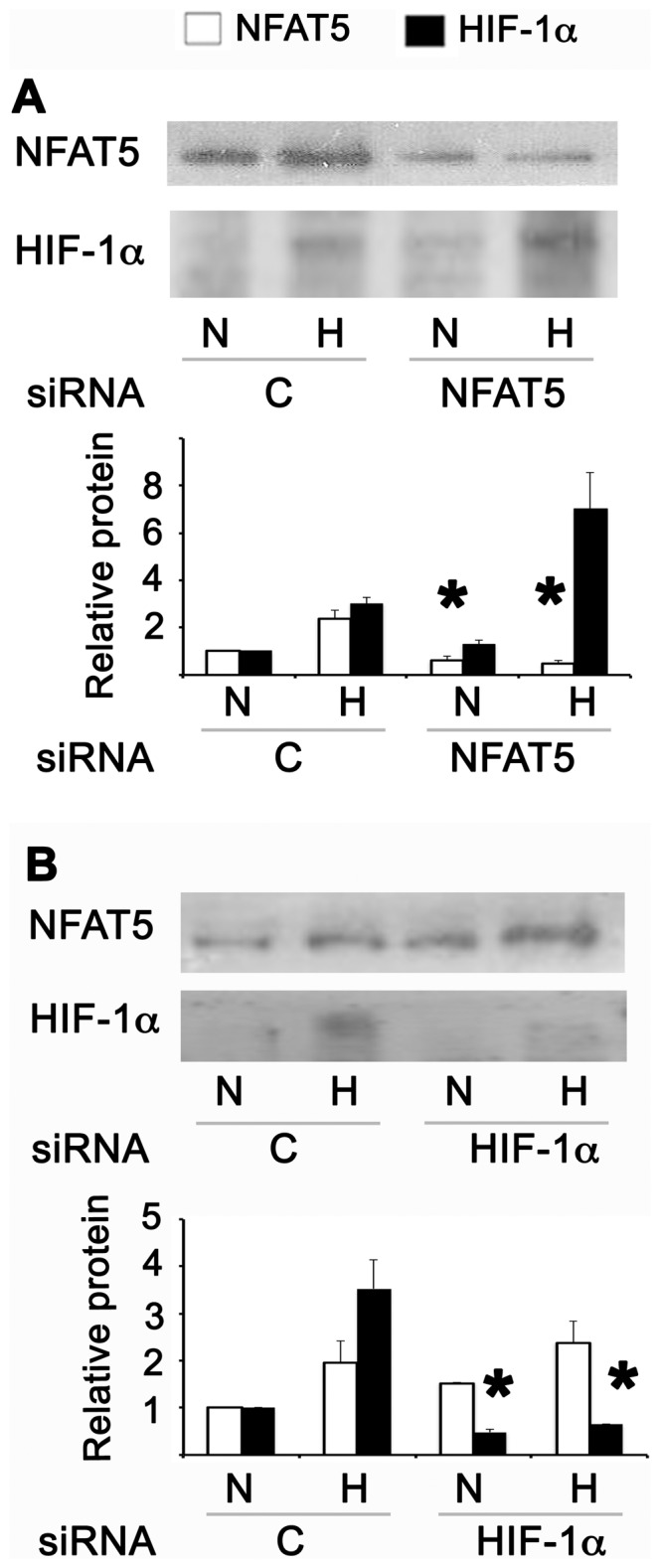
NFAT5 and HIF-1α are independently up-regulated by hypoxia. HEK293 cells cultured at 300 mosmol were transfected with control (C), NFAT5 or HIF-1α siRNA. 48 hrs after transfection the cells were cultured in normoxia (N) or 8 hrs of hypoxia (H). **A.** Protein abundance of NFAT5 and HIF-1α were studied by Western blot in cells transfected with siRNA against NFAT5. **B.** Protein abundance of NFAT5 and HIF-1α were studied by Western blot in cells transfected with siRNA against HIF-1α. A representative picture is shown in the upper section. Bar graph represents Mean ± SEM. *, P<0.05; n = 5.

Due to HIF-1α peaking at 2.5% O_2_, we decided to use this oxygen condition to evaluate the time course of NFAT5 and HIF-1α induction by hypoxia. The mRNA and protein of HIF-1α were induced from 4 hrs of hypoxia, with a maximum at 8 hrs (Figure 2B). However, the induction of NFAT5 mRNA and protein started only after 8 hrs of hypoxia and continued to increase at 16 hrs (Figure 2B). The earlier induction of HIF-1α suggested a potential regulatory role of HIF-1α on NFAT5 activation by hypoxia. To answer this question, we used siRNA against NFAT5 or HIF-1α in HEK293 cells. Knocking down the expression of HIF-1α did not affect the induction of NFAT5 in response to hypoxia (Figure 3A). Furthermore, siRNA knockdown of NFAT5 did not prevent the induction of HIF-1α by low oxygen (Figure 3B). These results establish that hypoxia can activate these two transcription factors by independent signalling pathways.

**Figure 4:**
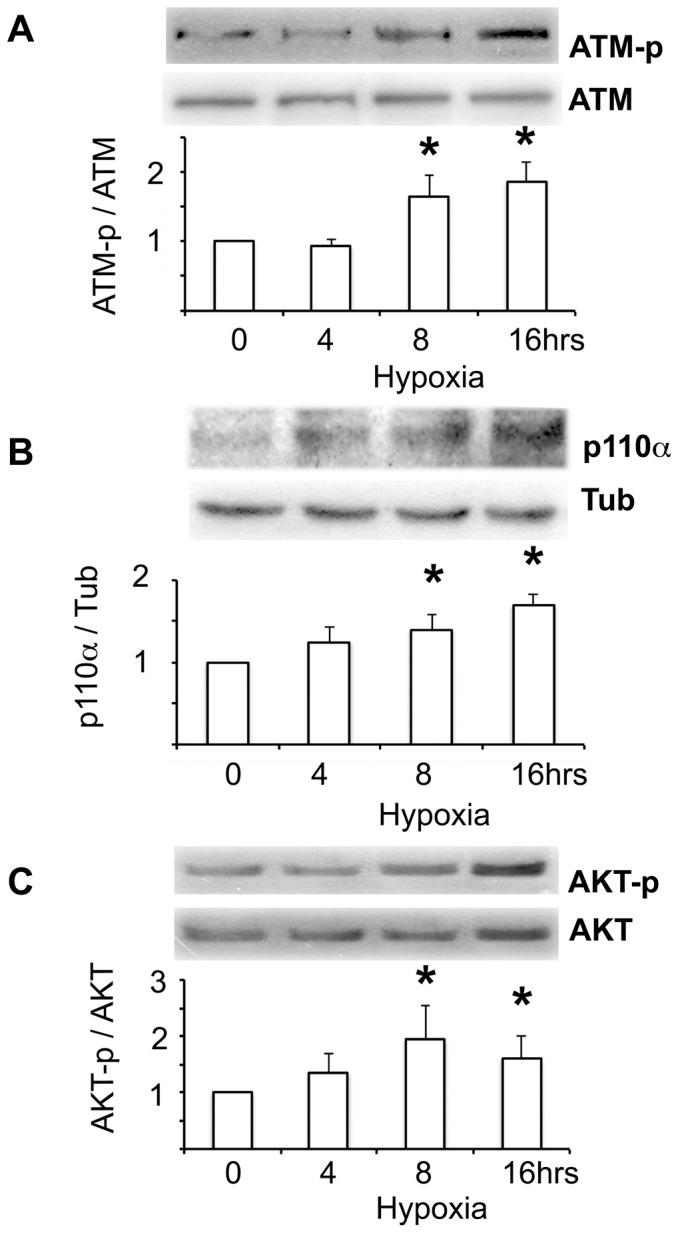
NFAT5-regulators protein, ATM and PI3K, were induced in HEK293 cells by hypoxia. HEK293 cells cultured at 300 mosmol were exposed for 0, 4, 8 and 16 hrs to hypoxia (2.5% of PO_2_). **A.** Time course response of ATM phosphorylation (normalized by total ATM) was measured by Western blot. **B.** Time course response of PI3K activation was measured by Western blot of HIF-1α protein abundance (normalized by tubulin: Tub). **C.** AKT-308 phosphorylation (normalized by total AKT) was measured by Western blot. A representative picture is shown in the upper section. Bar graph represents Mean ± SEM. *, P<0.05; n = 5.

**Figure 5:**
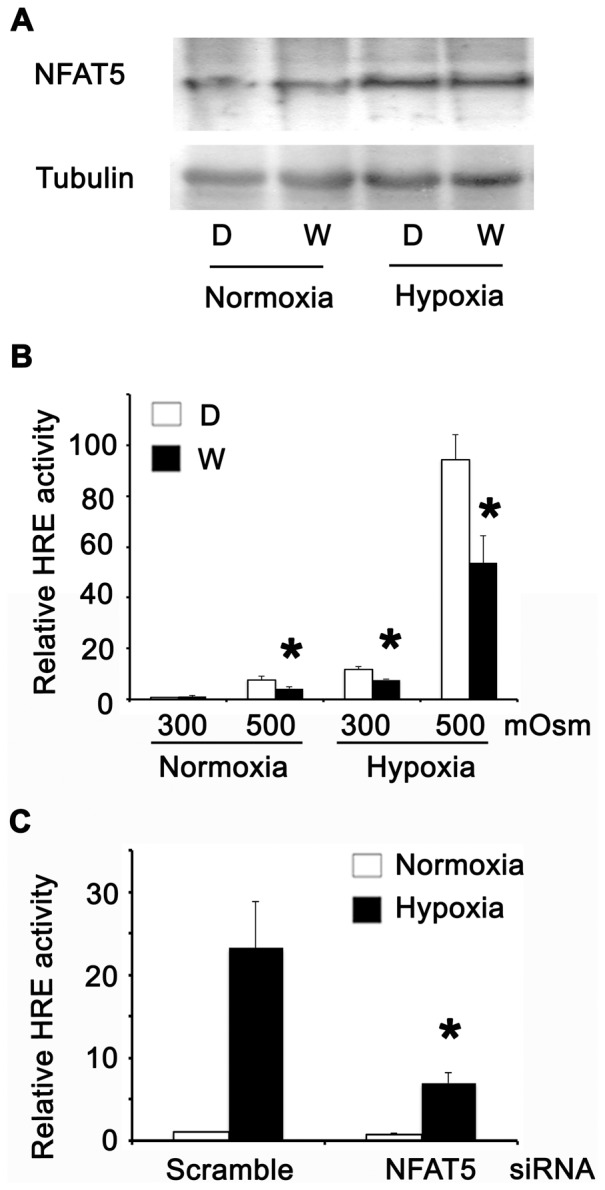
Wortmannin inhibited the NFAT5 activation by hypertonicity and hypoxia in HEK293 cells. **A.** Cultures at 300 mosmol were incubated with DMSO (D) or Wortmannin (W) by 1 hour. Then the cells were cultured in normoxia (N) or 8 hrs of hypoxia (H) and NFAT5 abundance was studied by Western blot. **B.** Cells cultured at 300 mosmol were transfected with HRE-Luciferase and 24 hrs after transfection the cells were incubated with DMSO (D) or Wortmannin (W) by 1 hour. After this treatment, the cells were cultured in normoxia (300 or 500 mOsM) or 8 hrs of hypoxia (300 or 500 mOsM) and the luciferase activity was assayed. **C.** Cells cultured at 300 mosmol were cotransfected with HRE-Luciferase and siRNA (control or NFAT5). 48 hrs after transfection the cells were cultured in normoxia or 8 hrs of hypoxia (300 or 500 mOsM) and the luciferase activity was assayed. Bar graph represents Mean ± SEM. *, P<0.05; n = 5.

Next, we studied potential signalling pathways that could modulate the activation of NFAT5 by hypoxia. In previous studies, we demonstrated that ATM and PI3K (p110α) are two positive regulators of NFAT5 in response to high NaCl [24], [25]. HEK293 cells exposed to hypoxia showed a time-dependent activation of ATM, as shown, by increased ATM phosphorylation after 8 and 16 hrs of hypoxia (2.5% O_2_) (Figure 4A). In addition to ATM, PI3K was activated by hypoxia in HEK293 cells. We observed a significant rise in the abundance of the p110α protein and AKT-308 phosphorylation after 8 hrs of hypoxia (Figure 4B and 4C). These results suggested that both kinases could be implicated in the activation of NFAT5 by hypoxia. However, in HEK293 cells transfected with HRE-luciferase reporter, the pharmacological inhibition of ATM and PI3K with 1 uM of Wortmannin did not prevent the protein induction of NFAT5 by hypoxia (Figure 5A). In contrast, Wortmannin reduced the HRE activation by hypertonicity, hypoxia or combined stimuli (Figure 5B). Knocking down NFAT5 we validate the participation of NFAT5 in the HRE activation (Figure 5C).

**Figure 6:**
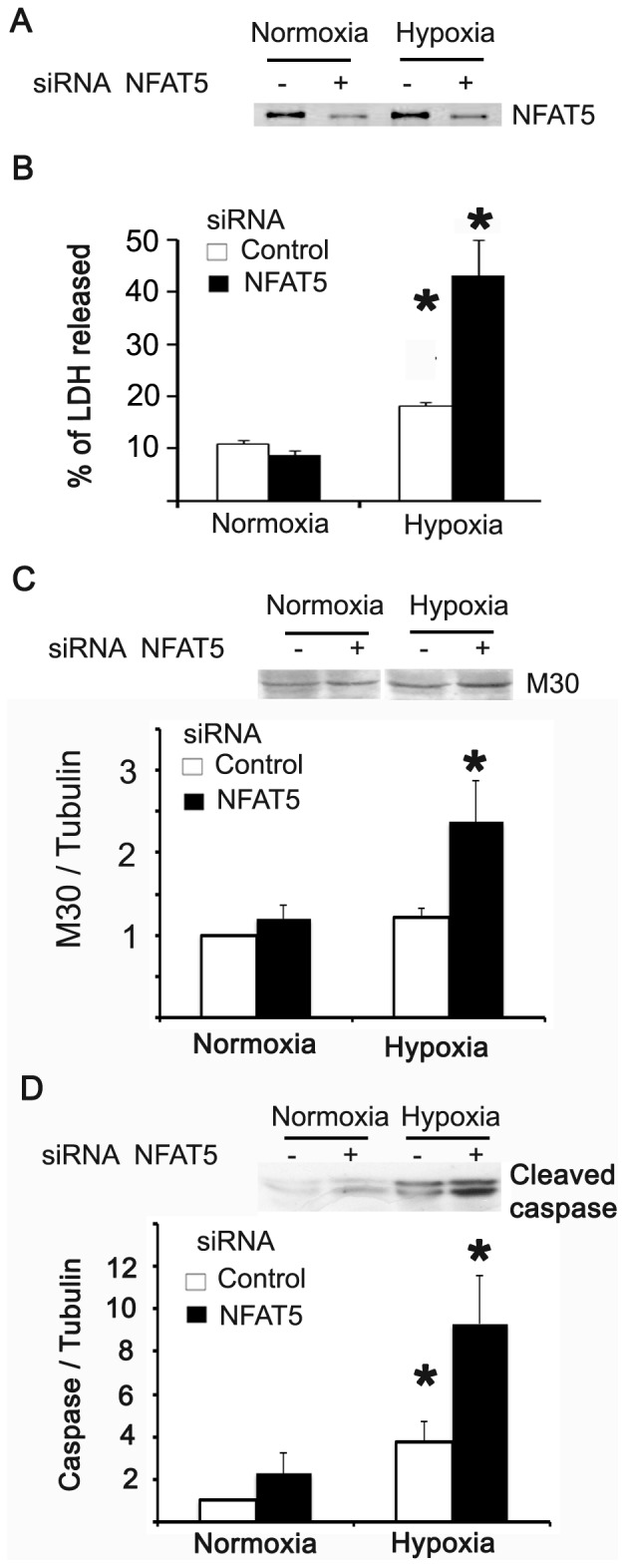
NFAT5 has a protective role against hypoxia. HEK293 cells cultured at 300 mosmol were transfected with control and NFAT5 siRNA. 48 hrs after transfection the cells were exposed for 8 hrs to 2.5% PO_2_. **A.** Western blot of NFAT5. **B.** LDH activity was assayed in cell culture media and cell lysate by spectrometric determination of NADH. **C.** Western blot of M30. **D.** Western blot of Cleaved caspase-3. Bar graph represents Mean ± SEM. *, P<0.05; n = 5.

Since NFAT5 is a pro-survival factor in osmotic stress, we investigated the role of NFAT5 in cell tolerance to hypoxia. The transient transfection of HEK293 cells with siRNA against NFAT5 decreased the abundance of this transcription factor, as compared with cells transfected with scrambled siRNA (Control, Figure 6A). We evaluated cell death by measuring lactate dehydrogenase (LDH) activity in the culture media. LDH activity was increased in hypoxic cells (2.5% O_2_, Figure 6B). The knockdown of NFAT5 by siRNA increased LDH activity (Figure 6B). To estimate apoptosis induced by hypoxia in HEK293 cells transfected with NFAT5/scrambled siRNA, we measured cleaved caspase-3 and M-30 in HEK293 cells. Active caspases 3 and 9 target the K8/18 proteins of the cellular intermediate filaments network, and the M30 CytoDeath monoclonal antibody recognizes a neo-epitope exposed after the cleavage of K18 by caspases. In the apoptotic cascade, these events precede the loss of membrane asymmetry and DNA fragmentation. Cleaved caspase-3 and M-30 were increased after 8 hours of hypoxia in control-transfected cells; when NFAT5 was knocked down and cells were exposed to hypoxia, these two apoptotic markers were induced (Figure 6C, D). These results suggest that NFAT5 has a protective role against cell death induced by hypoxia.

### NFAT5 expression in kidney exposed to experimental I/R

**Figure 7:**
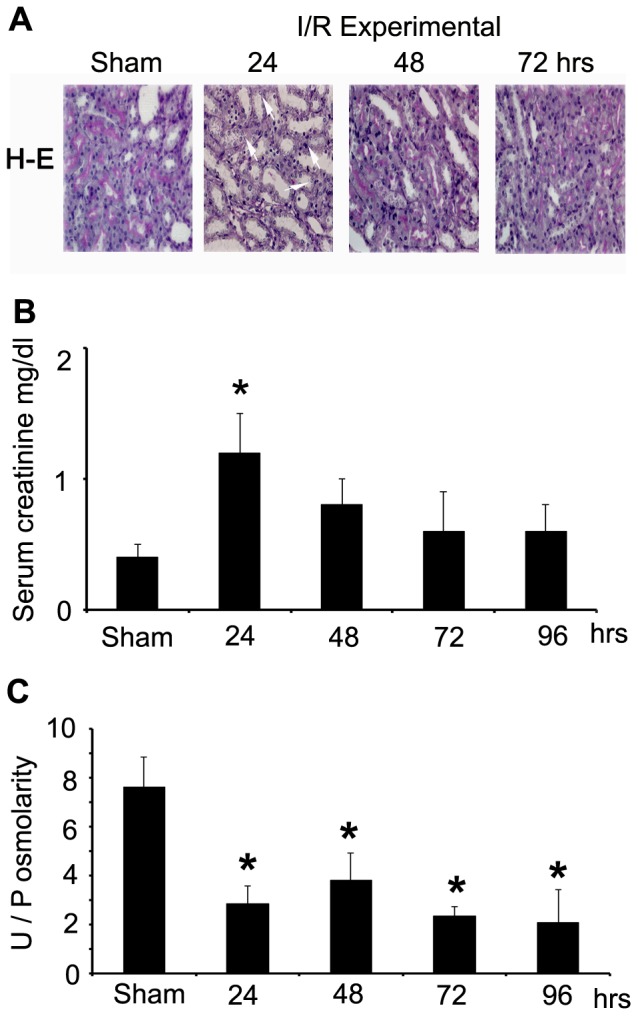
Kidney function after experimental I/R. **A.** Tissue damage evaluated by PAS staining. Brush border, epithelial flattening and mitosis were present in kidneys from I/R animals (arrows). **B.** Serum creatinine (mg/dl) of sham and 24–96 hrs post-ischemia. **C.** Urine and plasma ratio (U/P) of osmolality of sham and 24–96 hrs post-ischemia. Bar graph represents Mean ± SEM. *, P<0.05; n = 5.

We used morphological and functional analysis to evaluate kidney injury in an experimental model of I/R in rat. Kidney sections stained with PAS showed alterations in kidney morphology from 24 hours after I/R (Figure 7A). The most evident alterations were brush border flattening of the epithelia and a higher number of cells undergoing mitosis. These alterations were almost undetectable at 96 hours after I/R (data not shown). The I/R animals had higher serum creatinine levels than sham animals after 24 h hours of I/R (1.2 mg/dl; Figure 7B), which recovered after 96 hrs. Urine concentration, as estimated by determination of the U/P osmolality ratio, showed that experimental I/R animals excreted less concentrated urine than sham animals from 24 to 96 hrs of I/R (Figure 7C).

**Figure 8:**
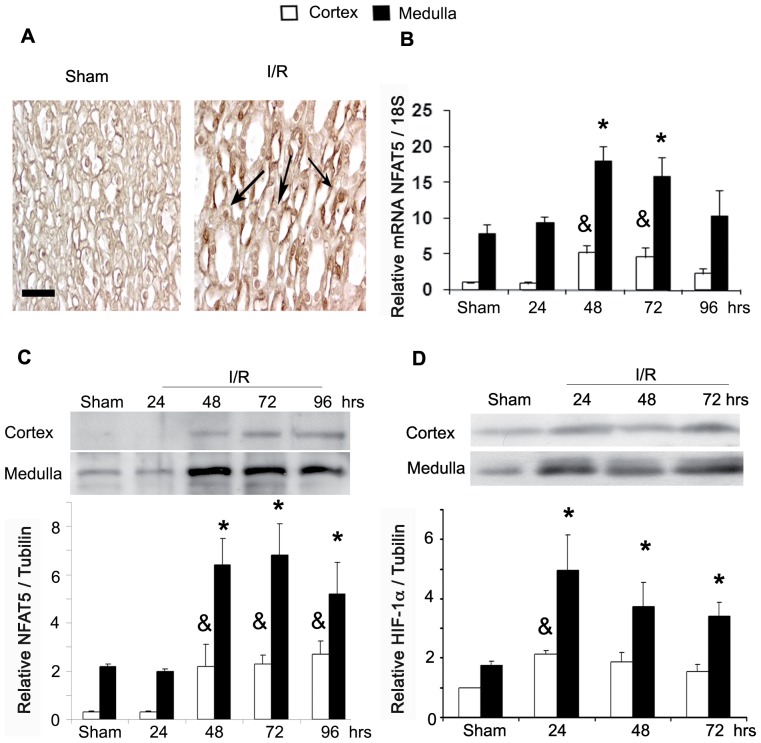
NFAT5 and HIF-1α are induced in post ischemic kidneys. **A.** Kidney sections of sham and I/R animals (72 hrs) were incubated with rabbit anti-NFAT5. Representative pictures of medulla from sham and I/R animals are shown. Preimmune serum did not stain significantly (data not shown). Scale bar = 100 µm. The arrows indicate the localization of the corresponding marker for NFAT5. **B.** NFAT5 mRNA abundance in cortex and medulla of kidneys were determined by qRT-PCR. **C.** NFAT5 protein abundance in cortex and medulla of kidneys were determined by Western blot **D.** HIF-1α (protein abundance in cortex and medulla of kidneys was determined by Western blot. A representative picture is shown in the upper section. Bar graph represents Mean ± SEM. * or & indicates P<0.05; n = 5 (* *vs* sham medulla and & *vs* sham cortex).

**Figure 9:**
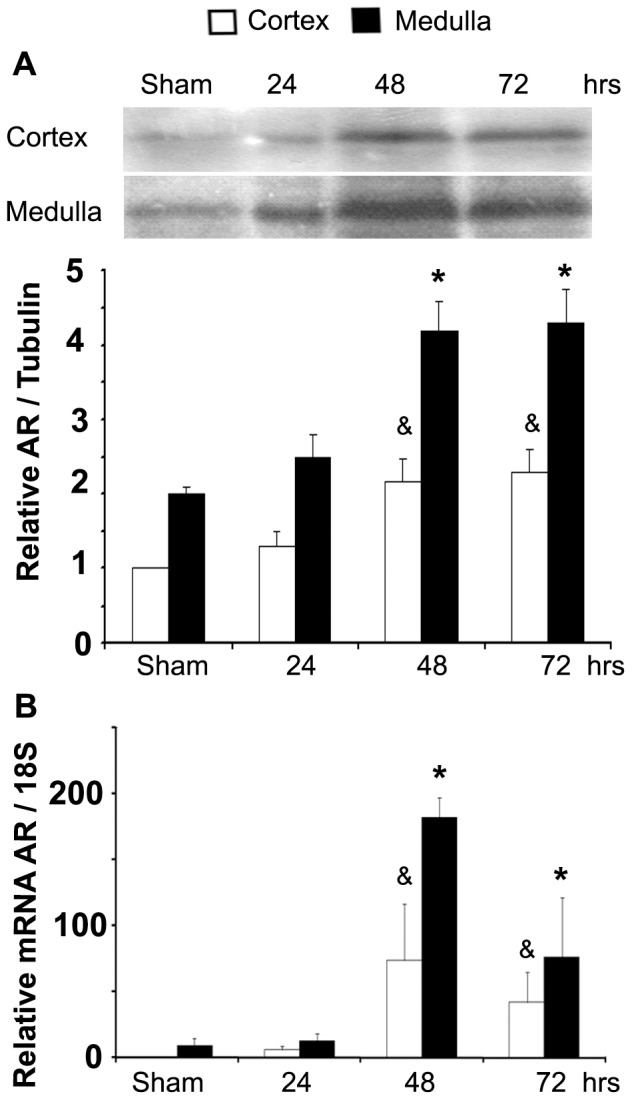
Experimental I/R induced renal Aldose Reductase (AR) expression. **A.** AR protein abundance in protein homogenates from cortex and medulla of rat kidney determined by Western blot. A representative picture is shown in the upper section. **B.** AR mRNA abundance in kidney cortex and medulla measured by qRT-PCR. Bar graph represents Mean ± SEM. * or & indicates P<0.05; n = 5 (*vs sham medulla & *vs* sham cortex).

The hypoxia response activation was demonstrated previously, using the same model of experimental I/R in rat kidney (2-pimonidazole and HIF-1α) [33]. NFAT5 expression was evaluated in kidneys from sham and experimental I/R animals by immunohistochemistry, qRT-PCR and Western blot. Immunohistochemical analysis of NFAT5 showed higher immunoreactivity in the renal medulla of I/R animals as compared with sham (Figure 8A). The signal was increased in the nuclei of tubular and blood vessel cells (Figure 8A; see arrows), suggesting NFAT5 activation in the kidney induced by ischemia and reperfusion. Using qRT-PCR and Western blot, we studied the time-course activation of NFAT5 in the kidneys of sham and I/R animals. At 24 hrs, kidneys of experimental I/R animals did not have differences in NFAT5 mRNA and protein abundance, as compared with sham animals. However, after 48 hours of reperfusion the abundance of NFAT5 protein (Figure 8B) and mRNA (Figure 8C) was significantly increased in cortex and medulla. We also evaluated HIF-1α protein abundance in kidneys from experimental I/R and the results showed significant induction of HIF-1α, both in cortex and medulla (Figure 8C), peaking after 24 hrs of reperfusion. HIF-1α induction was earlier than NFAT5. To evaluate NFAT5 activity in kidneys of experimental I/R animals, we analysed the NFAT5-target gene, Aldose Reductase (AR). In basal conditions AR mRNA and protein abundance was higher in the medulla than the cortex of sham animals (Figure 9). I/R increased AR expression, both in the cortex and medulla of kidneys with similar NFAT5 induction timing (Figure 8).

**Figure 10:**
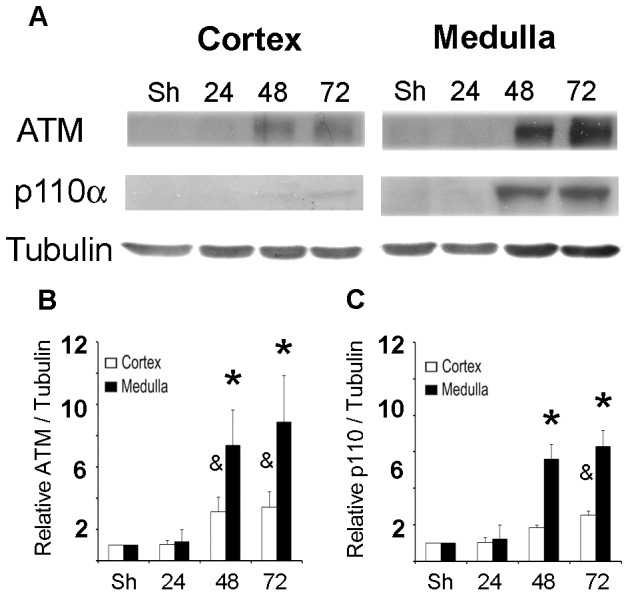
NFAT5-regulators protein, ATM and PI3K, were induced in post-ischemic kidneys. ATM and PI3K (p110α) protein abundance was measured by Western blot in cortex or medulla from rat kidney. A. A representative picture is shown in the upper section (n = 5). **B.** Relative ATM abundance. **C.** Relative p110α abundance. Bar graph represents Mean ± SEM. * or & indicates P<0.05; n = 5 (* *vs* sham medulla & *vs* sham cortex).

Finally, we studied ATM and PI3K (p110α) protein abundance *in vivo* (experimental I/R). Kidneys from I/R animals showed increased ATM and p110α protein abundance in the cortex and medulla (Figure 10). Both proteins were increased from 48 hrs of I/R according with the NFAT5 induction (Figure 8B, C). However, p110α was weakly detectable in renal cortex (Figure 10).

## Discussion

### Upregulation of NFAT5 by low PO_2_

Our results showed that anoxia/hypoxia (0–2.5% oxygen) induced NFAT5 protein abundance in IMCD and HEK293 cells. NFAT5 induction under hypoxia was observed when epithelial cells were cultured in isotonic (300 mOsM) and hypertonic media (500 or 640 mOsM). We observed that hypertonicity and hypoxia had additive effects in the induction of NFAT5. All these data suggest that low PO_2_ is a positive regulator of NFAT5 expression, independent of the hypertonicity.

In addition to the increased abundance of NFAT5 caused by anoxia (0% O_2_) in cultured IMCD cells, we observed the nuclear translocation of NFAT5. Similarly, using HEK293 cells, we found that hypoxia (2.5% O_2_) induced nuclear translocation and activation of transcription activity of NFAT5 (OREX and HRE-luciferase). The activation of NFAT5 by hypoxia was independent of HIF-1α. All these results indicate that low PO_2_ not only increased the protein abundance of NFAT5, but also triggered its transcriptional activity without the participation of HIF-1α.

Our studies in HEK293 cells showed that knocking down the expression of NFAT5 did not affect cell death in isotonic-normoxic conditions. However the reduction in NFAT5 expression caused an important increase in apoptosis and necrosis after 8 hours of hypoxia. These data indicate that NFAT5 has a previously unidentified protective role against hypoxia. Medullary hypoxia is a price that the mammalian kidney pays for efficient urinary concentration [34], [35], and NFAT5 in renal medulla may improve tolerance for hypertonicity in a hypoxic context. Further studies will be required to test this hypothesis, and to establish the potential protective role of NFAT5 against hypoxic insults in other cells and tissues.

### Upregulation of NFAT5 in experimental I/R

To test if hypoxia is a positive regulator of NFAT5 *in vivo*, we used experimental I/R, induced by temporary bilateral renal ischemia for 30 min. Experimental I/R animals have significantly increased plasma creatinine levels and reduced U/P osmolality, indicating acute kidney injury (AKI) (Figure 7). Our results showed a strong upregulation of NFAT5 protein expression in both cortex and medulla of post-ischemic kidneys (Figure 8). The induction of NFAT5 was slower than the induction of HIF-1α, but persisted up to 72 hours post-ischemia. The impairment of urinary concentration ability (reduced U/P osmolality) in experimental I/R animals has been associated with a decreased level of aquaporins (both in the proximal tubule and collecting duct of post-ischemic kidneys) [36] and sodium transporters [Na, K-ATPase, rat type 1 bumetanide-sensitive Na-K-2Cl cotransporter (BSC-1), Na/H exchanger type 3 (NHE3), and thiazide-sensitive sodium chloride cotransporter (TSC)] [37]. Our *in vivo* data indicate that transient hypoxia up-regulated NFAT5 expression in isotonic conditions (cortex) and in the medulla of I/R kidney. The induction of NFAT5 under hypoxic conditions *in vitro* and *in vivo* observed in the present study supports the conclusion that low PO_2_ is an independent upregulator of NFAT5 in kidney cells and that it could have a protective role.

Consistent with our studies in cultured cells, immunoreactivity of NFAT5 was found in the nucleus of tubules of post-ischemic kidneys, suggesting that NFAT5 protein up-regulation is associated with increased transcriptional activity. Furthermore, the NFAT5 up-regulation in post-ischemic kidneys was associated with increased expression of AR (mRNA and protein) (Figure 9), indicating increased NFAT5 activity.

### Mechanism of NFAT5 activation by hypoxia

In the present study, exposure of HEK293 cells to hypoxia caused a rapid increase in HIF-1α and NFAT5 (mRNA and protein), but HIF-1α was induced early. However, NFAT5 was translocated to the nucleus at 2 hrs of hypoxia, suggesting early activation of NFTA5, independent of the induction of its gene expression. Moreover, severe hypoxia (1% oxygen, Figure 2A) produced the highest levels of NFAT5, but not for HIF-1α. Sorbitol-treated AT2 cells demonstrated induction of HIF-1α by hypertonicity [38], suggesting that both NFAT5 and HIF-1α could respond to hypoxia and hypertonicity. Despite this information we did not find HIF-1α induction by 500 mosmol (4–16 hrs) in HEK293 cells (data not shown). Although the analysis of 3000 bp upstream to the initiation site of the NFAT5 gene (human, homo sapiens, gene ID: 10779) showed one hypoxia-response element (HRE) consensus site, our experiments using siRNA knockdown of NFAT5 or HIF-1α suggest that hypoxia induces both transcription factors by different signalling pathways.

Our results in post-ischemic kidneys demonstrated that both ATM and PI3K (p110α) were induced in I/R kidneys (Figure 10). We also found ATM and PI3K activation in HEK293 cells exposed to hypoxia (2.5% oxygen) (Figure 4). Using pharmacological inhibition (Wortmannin), we found a potential role of these kinases in the NFAT5 activation by hypoxia (Figure 5). ATM activation has also previously been described during exposure to hypoxia [31], [39], [40]. Recent studies in cancer cells demonstrate alternate mechanisms for activating ATM under hypoxic conditions, including the increase of radical oxygen species, oxidative stress and DNA breaks [41]. In cancer cells exposed to hypoxia, ATM remains diffuse throughout the nucleus, as does phosphorylated ATM [32]. This localization pattern is reminiscent of that seen in response to high salt [32], indicating that hypoxia and osmotic stress may share a similar mechanism of ATM activation. So, our results and others demonstrate ATM and PI3K activation by hypoxia, suggesting its participation in NFAT5 activation by this stimulus.

NFAT5 belongs to the nuclear factor of activated T-cells (NFAT). NFAT proteins were originally defined as calcium/calcineurin-dependent regulators of cytokine gene transcription in T lymphocytes. NFAT5 can be induced in both primary quiescent T lymphocytes and differentiated Th1 and Th2 cell populations upon mitogen- or antigen receptor-dependent activation [42]. However, induction of NFAT5 by a hyperosmotic stimulus in cultured epithelial cells is not blocked by the inhibition of calcineurin [42] Further studies are needed to clarify if hypoxia activates calcineurin-dependent mechanisms for NFAT5 induction/activation in kidney cells.

Recently studies have shown that nitric oxide is increased in I/R in kidneys [43]–[45] and some reports suggest that NO could counteract NFAT5 activation. It is possible that NO could participate in this phenomena. We did not measure the nitric oxide concentration.

In summary, the present study showed sufficient evidence to propose NFAT5 activation by hypoxia in kidney epithelial cells and its potential protective role against hypoxia. Our results encourage further study of the role of this transcription factor in hypoxia-induced kidney damage induced as well as its contribution in other tissues exposed to hypoxia and ischemia/reperfusion.

## Materials and Methods

### Animals

Adult male Sprague-Dawley rats (250 g, n = 5 for each I/R group: 24 h, 48 h, 72 h and 96 hrs) were housed in a 12 h light/dark cycle. Animals were weighed at the time of initiation of bilateral ischemic injury and after completion of experiments. The animals had food *ad libitum* and controlled water and were maintained at the University animal care facility. All experimental procedures were in accordance with institutional and international standards for the humane care and use of laboratory animals (Animal Welfare Assurance Publication A5427-01, Office for Protection from Research Risks, Division of Animal Welfare, The National Institutes of Health). The Committee on the Ethics of Animal Experiments of the University de Los Andes number 1908–09 approved the protocol.

### Renal ischemia/reperfusion injury

Animals were anesthetized with ketamine∶xylazine (25∶2.5 mg/kg, ip), maintaining a body temperature of 37°C. Both kidneys were exposed by a flank incision, and both renal arteries were occluded with a non-traumatic vascular clamp for 30 minutes. After 30 minutes of clamping, clamps were removed, renal blood flow was re-established, both incisions were sutured, and rats were allowed to recover in a warm room. Rats were euthanized under anesthesia (ketamine∶xylazine) 24, 48, 72 and 96 hrs after reperfusion; both kidneys were removed and processed for immunohistochemistry, real- time PCR and Western blotting [27], [33]. A group of sham animals were included; the kidneys of these animals were exposed by a flank incision, but they did not receive renal artery occlusion.

### Cell Culture and Treatment

Primary cultured cells were obtained from the inner renal medulla of male Sprague-Dawley rats (120–150 g body weight). The papillary tissue was finely minced with a surgical blade under sterile conditions. The tissue was digested into a 10 ml culture medium (DMEM/Ham's F12, 5 mg/ml transferring, 5 mg/ml human insulin, 50 nM hydrocortisone, 5 pM triiodothyronin, 50 UI/ml penicillin and 50 mg/ml streptomycin) plus 20 mg collagenase and 7 mg hialuronidase at 37°C in a shaker for 90 minutes. After the tube was centrifuged at 1000 rpm for 1 minute, the supernatant was discarded and the pellet was suspended in DMEM/Ham's F12. This procedure was repeated 3 times. Finally, the pellet was suspended in culture medium with 10% fetal bovine serum (FBS) and cultured in a 30 mm culture dish. Cells were incubated at 37°C in a 5% CO2 atmosphere. HEK293 cells and HEK293 cells stably expressing ORE-X (HEK293-OREX) [27] were cultured in a 300 mosmol/kg medium according to ATCC (American Type Culture Collection, Manassas, VA) instructions. Wortmannin was dissolved in DMSO and the cells were incubated with 1 µM by 1 hr.

### Hypoxia in cell cultures

Cultured cells were incubated in isotonic (300 mOsM) or hypertonic (500 or 640 mOsM) media for 24 hrs at normoxic condition (21% O_2_). NaCl was added to the isotonic culture media to make the solutions hypertonic. During the 24 hrs of culture in isotonic or hypertonic media, cells were incubated in anoxic/hypoxic (0–5% O_2_) conditions for time course experiments (0, 4, 8 or 16 hours at 37°C). Hypoxic conditions were obtained by replacing oxygen with N_2_, using a Heracell 150i CO_2_ incubator (Thermo Scientific). To test the oxygen percentage in the incubator, we used the Multi-Gas Detector, model Dräger X-am® 2000 (Lübeck, Germany).

### Plasmids and siRNAs

The ORE-X Luciferase reporter construct contains two copies of human ORE-X, within a minimal IL-2 promoter [17]. The HRE-Luciferase is a pGL2 vector containing three hypoxia response elements from the Pgk-1 gene [46]. The siRNAs were designed as a synthetic dsRNA Dicer substrate to enhance the RNA interference potency and efficacy. The control and NFAT5 siRNAs have been described previously [26], [47]. The siRNA against to HIF-1α Duplex sequences were: sense, 5′-Phos-GAAGGAACCUGAUGCUUUAACUUdTdG-3′ and antisense 5′- CAAAGUUAAAGCAUCAGGUUCCUUCUU-3′ (Integrated DNA Technologies, Coralville, IA).

### Luciferase Assays

HEK293 cell were cultured as described above. The culture solution was harvested and the supernatant was recovered by centrifugation at 12000 rpm. HEK293-OREX cells [19], [24], [25] and HEK293 cells transient transfected with HRE [46] were cultured in isotonic (300 mOsM) or hypertonic media (640 mOsM) for 24 hrs. In both osmotic conditions, the cells were exposed to anoxia (0, 8 or 16 hours at 37°C) as described above. Lysis reagent (CCLR) was added into each culture vessel. The attached cells were scraped and centrifuged to 12,000 rpm×15 seconds, and the supernatant was transferred to a new tube. Luciferase activity was measured with the Luciferase Assay System (Promega, Madison, WI) using the Biotex Luminometer. Luciferase activity was expressed in relative light units (RLU) per µg of total cell protein.

### Lactic Dehydrogenase (LDH) activity

The reaction velocity was determined by a decrease in absorbance at 340 nm resulting from the oxidation of NADH using the Spectrophotometer: GeneQuant™ 1300 (GE Healthcare). One unit causes the oxidation of one micromole of NADH per minute at 25°C and pH 7.3. Into 0.9 ml of reaction mix [Tris⋅HCl, (0.2 M, pH 7.3), NADH (6.6 mM, pH 7.3) and Sodium pyruvate (30 mM, pH 7.3)] was added 0.1 ml of appropriately diluted sample and recorded the ΔA340/min from initial linear portion. The results were expressed as % of LDH released.

### Western Blot Analysis

Total protein was measured using the BCA Protein Assay Kit, (Pierce, Rockford, IL). Cortex and medulla kidney sections from one half kidney were homogenized with an Ultra-Turrax homogenizer in lysis buffer containing 50 mMTris-HCl, pH 8.0, 150 mM NaCl, 1% Triton X-100, protease inhibitor (Complete Mini, Roche Applied Science, Indianapolis, IN), and phosphatase inhibitor cocktails (Phosphatase Inhibitor Cocktails 1 & 2, Sigma, St. Louis, MO). Tissue homogenates were then centrifuged (13,000× g, 10 min) and the supernatant was stored (−80°C) for SDS_PAGE and Western blot analysis. Proteins were separated on 7.5 or 10% Tris-Glycine gels and transferred to nitrocellulose membranes (Invitrogen, Carlsbad, CA). Western blot analysis was performed according to standard conditions [19]. In brief, after blocking nonspecific binding, membranes were incubated with rabbit anti-NFAT5 (NFAT5) (Affinity Bio Reagents, Golden, CO), goat anti-AR (Santa Cruz Biotechnology, Santa Cruz, CA), rabbit anti-tubulin (Cell Signalling), rabbit anti-cleaved Caspase-3 (Asp175) (Cell Signalling), mouse anti-M30 CytoDEATH (ROCHE), rabbit anti-ATM (cell signalling), rabbit anti-phospho ATM (cell signalling), rabbit anti-p110α (Cell signalling), rabbit anti-AKT (Cell signalling), rabbit anti-phospho-AKT-308 (Cell signalling) or mouse anti-HIF-1α (Abcam) antibody overnight at 4°C. After washing with 0.1% Tween-20 in PBS, blots were incubated with the appropriate horseradish peroxidase (HRP)-conjugated secondary antibody for 1 hour at room temperature. Proteins were detected using an enhanced chemiluminescence technique (PerkinElmer, Life Sciences, Boston, MA). The blots were scanned and densitometric analysis was performed using the public domain NIH Image program v1.61 (US National Institutes of Health, http://rsb.info.nih.gov/nih-image).

### Fluorescence microscopy

First passage rat IMCD cells were grown in 8-well Chamber-Slides (Lab-Tek, Nunc). After 24 hours of culture in isotonic (300 mOsM) media, the tissue culture slides were incubated for 2 hrs in anoxia (O_2_ replaced by N_2_, Heracell 150i CO_2_ incubator; Thermo Scientific). After this time the culture medium was quickly removed, the fixing reagent was added (100% of cold methanol) and cells were stored overnight at −20°C. Then, the cells were washed, incubated in the presence of blocking solution (1 hour, room temperature) and incubated with anti-NFAT5 antibody (1 hour, room temperature) (Affinity BioReagents, Golden, CO). After washing, the secondary antibody was added (1 hour Alexa 488-Green, 1∶200 dilution, Invitrogen). Nuclei were stained with Hoechst 33258 (Sigma). The slides were mounted and NFAT5 cellular distribution was analyzed by fluorescence microscopy using an Olympus BX61WI upright microscope with an Olympus DSU spinning disk unit. Images were recorded with the cooled charge-coupled device video camera (SIS-FVT2, OLYMPUS) and analyzed using the imaging software (CellM&CellR, OLYMPUS).

### Immunohistochemical analysis and tissue damage determination

Immunohistochemical studies in paraplast-embedded sections were carried out by tissue processing according to previously described methods. Briefly, tissue sections were dewaxed, rehydrated, rinsed in 0.05 M tris-phosphate-saline (TPS) buffer (pH 7.6) and incubated with rabbit anti-NFAT5 antibody (Affinity BioReagents, Golden, CO) overnight at 22°C. Afterwards, sections were washed three times for 5 minutes each, followed by 30 minute incubation at 22°C with the corresponding secondary antibody and with the peroxidase-antiperoxidase (PAP) complex. Immunoreactive signals were revealed using 3,3′-diaminobenzidine 0.1% (wt/vol) and 0.03% (vol/vol) hydrogen peroxide solution. Periodic acid-Schiff (PAS) staining was used to determine tissue damage.

### Real Time PCR

Total RNA was isolated using TRIzol (GIBCO, Life Sciences) as per manufacturer instructions. RNA concentration was determined by spectrophotometry and integrity of the RNA was assessed by agarose gel electrophoresis. cDNA was prepared from total RNA (0.5 µg) using a reverse transcription system (random hexamers, Improm II Reverse Transcriptase System; Promega). PCR was performed on 8 ng and 80 ng cDNA samples per 20∶l reaction in triplicate for each experiment (GoTaq Flexi DNA polymerase, Promega). Amplicons were detected for Real-Time Fluorescence Detection (Rotor-Gene Q, Qiagen). Primers used to qPCR were to aldose reductase (AR) were 5′-ATTCGTCCACCACAGCTTCAGACT-3′ and 5′- AGCAATGAGGACATGGCCACTCTA-3′; NFAT5 5′-TTCATCTCATTGCTCAGCG-3′ and 5′-GGGAGAAGATCATAGACAGATTC-3′; HIF-1α 5′-ACCTCTGGACTTGCCTTTC-3′ and 5′-TTTTTCTTGTCGTTCGCGC-3′. and 18S (housekeeping) 5′-TTAGAGTGTTCAAAGCAGGCCCGA-3′ and 5′-TCTTGGCAAATGCTTTCGCTCTGG-3′. The detection system records the number of PCR cycles (Ct) required to produce an amount of product equal to a threshold value, which is a constant. From the Ct values, we calculated the relative mRNA abundance in each experimental condition, and values were normalized to the relative abundance of each transcript in tissue from paired sham animals, as described [19].

### Statistical Analysis

Data are expressed as average ± SEM. Values from different groups were assessed with the parametric Student's t-test when comparing two groups and Anova for multiple comparisons with a post-hoc Fisher's test when comparing more than two groups. The significance level was p<0.05.
